# cellMCD Effectively Discovers Drug Resistance and Sensitivity Genes for Acute Myeloid Leukemia

**DOI:** 10.3390/genes17010049

**Published:** 2026-01-01

**Authors:** Dora Obodo, Nam H. K. Nguyen, Xueyuan Cao, Phani Krishna Parcha, Christopher D. Vulpe, Jatinder K. Lamba, Stanley B. Pounds

**Affiliations:** 1 Department of Biostatistics, St. Jude Children’s Research Hospital, Memphis, TN 38105, USA; dora.obodo@stjude.org; 2 Department of Pharmacotherapy and Translational Research, Center for Pharmacogenomics and Precision Medicine, College of Pharmacy, University of Florida, Gainesville, FL 32610, USA; namnguyen@ufl.edu (N.H.K.N.); phanikrishparcha@ufl.edu (P.K.P.);; 3 Department of Health Promotion, Disease Prevention, and Preventive Medicine, The University of Tennessee Health Science Center, Memphis, TN 38163, USA; xcao12@uthsc.edu

**Keywords:** multi-omic prognostic association studies, pediatric acute myeloid leukemia, CRISPR screens, cellMCD, Fisher’s method, SSz method

## Abstract

Background: Rapid advances in biotechnology provide researchers with the opportunity to integrate omics profiles (genomics, epigenomics, transcriptomics, proteomics, etc.) with multiple phenotypes or experimental conditions. In cancers such as acute myeloid leukemia (AML), where combination therapies are standard of care, identifying genetic drivers of drug resistance requires evaluating how genes are associated with multiple drug response phenotypes. Statistical analyses associating omics profiles with multiple phenotypes yield multiple significance values and rankings for each of many genes. There is a great need to consolidate these multiple rankings into a consensus ranking to prioritize specific genes for detailed follow-up wet-lab or clinical studies. Methods/Results: Here, we evaluate the well-known Fisher’s method, the sum of squared z-statistics (SSz), and the recently published cellMCD method as tools for gene prioritization. In simulation studies, cellMCD showed very similar or highly superior performance to the widely used Fisher’s and SSz methods. These advantages were also observed in an example application involving a CRISPR drug screen of an acute myeloid leukemia cell line. Conclusions: In summary, our results indicate that cellMCD should be more widely used for prioritizing discoveries from multiple omic association studies. These methods are available as an R package on github.

## 1. Introduction

New biotechnologies are powerful tools to query the molecular mechanisms driving human diseases. High-throughput nucleic acid sequencing technologies provide investigators with comprehensive data on the genome, epigenome, and transcriptome at the bulk tissue sample or individual cell level [1]. In cancer research, these technologies have been used to capture multi-omic profiles of diagnostic tumor tissue and associate those profiles with clinical outcomes [2]. These multi-omic prognostic association studies have provided valuable insights regarding the molecular drivers of prognosis.

Additionally, investigators may now use CRISPR-Cas9 gene editing technology to selectively introduce genetic alterations into cells and then evaluate the functional consequences of those alterations in cells with or without various drug treatments. CRISPR drug resistance screens simultaneously evaluate the impacts of thousands of gene knockouts on the growth of cells in the presence of one or more drugs [3]. CRISPR drug screens can provide mechanistic insights that complement the discoveries of genomic prognostic association studies.

CRISPR drug resistance screens and, more broadly, genomic prognostic association studies each produce high-dimensional statistical analysis results. CRISPR drug resistance screenings provide statistical analysis results for the acceleration or inhibition of growth of multiple cell lines with knockout of each gene under each of several treatments [3]. Prognostic association studies provide statistical analysis results associating the various omic measurements (genomic, epigenomic, transcriptomic, etc.) of each gene with multiple clinical outcomes (disease response, time to relapse, survival times, etc.) [4]. These studies may provide dozens of statistical analysis results for each of thousands of genes.

It is challenging to effectively translate these multi-dimensional statistical results into prioritized directions for future research. Several different patterns of statistical analysis results can be biologically and clinically meaningful for different reasons. In a CRISPR drug resistance screen, a multi-drug resistance gene promotes resistance to multiple drugs and thus may be a useful target to improve the efficacy of treatments involving those drugs. A gene may promote resistance to one drug but sensitivity to another and may indicate a synergistic or antagonistic pleiotropy [4]. A gene may promote resistance to a specific drug in some cell lines but sensitivity to that same drug in other cell lines; such a gene may be a candidate for defining an individualized treatment rule [5]. Furthermore, a gene may be associated with poor tumor response and patient outcomes in a genomic prognostic association study. The PROMISE [6] and CC-PROMISE [7] methods are designed to find features in one- and two-omic data matrices that exhibit specific scientifically interesting patterns of association with multiple phenotypes. These methods are powerful but require a fairly large number of subjects to have all forms of data because statistical significance is determined by permutation. It would be very valuable to have a tool that can identify multi-dimensional patterns of interest and provide a consensus ranking of genes to help prioritize further study without relying on permutation testing or requiring all forms of data to be available on a large number of subjects.

Here, we extend and evaluate the cellwise minimum covariance determinant (cellMCD) [8,9] method as a tool to identify genes that exhibit distinctive association patterns in a large matrix of z-statistics that associate genes (rows) with multiple phenotypes (columns). In this context, cellMCD may be used to detect row outliers, which are distant from the bulk of rows in multivariate space, and entry outliers, which have unusual values given the other values in the same row and the overall pattern of the bulk of the data. In this way, cellMCD can integrate information from multiple phenotype association analyses (each producing a column of z-statistics) to generate a consensus ranking of genes by row-outlier detection *p*-values and a consensus ranking of individual gene–phenotype associations by a standardized residual metric used for entry-outlier detection. In this work, we evaluate the performance of cellMCD for these two tasks in simulation studies and in an example CRISPR cell line drug resistance screening data set.

## 2. Fisher’s Method and the Sum of Squared z-Statistics

Let g=1,…,m index genes that are evaluated for each of j=1,…,d statistical associations. Suppose that each statistical association is summarized by a z-statistic $z_{gj}$ and let Z  represent the matrix of these z-statistics. Our objectives are to (1) identify specific entries of  Z that are outliers relative to the most common pattern of values observed in Z and (2) identify specific rows of Z that are outliers from the most common pattern of values observed in Z. Objective (1) will identify specific gene–phenotype associations that may be biologically interesting and worthy of follow-up research. Objective (2) will identify specific genes that exhibit unique patterns of association with one or more phenotypes and thus may be worthy of further study.

Fisher’s method for combining *p*-values is an easily implemented technique to compute a consensus *p*-value for each gene and generate a consensus ranking of genes [10]. Fisher’s method first computes a *p*-value $p_{gj\;}$ for each z-statistic $z_{gj},$ then computes $t_{g}=-\sum_{j=1}^{d}2ln(p_{gj})$ for each gene and compares it to a central chi-square distribution with  2d degrees of freedom to obtain a consensus *p*-value $p_{g}$. This consensus *p*-value may then be considered. A consensus ranking of genes is then obtained by ranking genes according to their consensus *p*-values. The results may be reported in terms of the consensus *p*-values and a standardized criterion $t_{g}^{*}=\sqrt{t_{g}/2d}$. A weighted version of Fisher’s method has recently been proposed as a powerful meta-analysis method [11].

The sum of squared z-statistics (SSz) is another easily implemented method to obtain a consensus *p*-value for each gene and generate a consensus ranking of genes. Cochran showed that the sum of squared independent and identically distributed standard normal observations follows a central chi-square distribution [12]. Thus, for each gene g, SSz computes $w_{g}=\sum_{j=1}^{d}z_{gj}^{2}$ and compares it to a central chi-square distribution with d degrees of freedom to obtain a consensus *p*-value. As before, the genes’ consensus *p*-values are used to produce a consensus ranking of genes. The results may be reported in terms of the consensus *p*-values and the root mean square z-statistics ${RMSZ}_{g}=\sqrt{w_{g}/d}$ as a standardized criterion.

Fisher’s method and SSz assume that for each gene, the z-statistics are independent under the null hypothesis. Violation of this assumption can inflate the statistical significance of the consensus *p*-values and thereby reduce the value of the consensus rankings for prioritizing genes for further study. Additionally, the consensus *p*-values and consensus rankings do not directly identify specific associations that drive genes to rank highly in the lists and do not directly evaluate the relative placement of genes in a multivariate space defined by ***Z***. Methods that are not subject to these specific limitations and assumptions may provide complementary biological insights that are missed by Fisher’s method and SSz. This motivated us to consider cellMCD as an alternative method to prioritize specific gene associations for further study and produce a consensus ranking of genes for further evaluations.

## 3. The cellMCD Method

The cellwise minimum covariance determinant (cellMCD; [8]) estimator is a procedure to estimate the center and covariance of a multivariate distribution that is robust against outliers in individual entries of the observed data matrix. The cellMCD procedure adapts a missing data multivariate normal likelihood criterion for the purpose of robust estimation and outlier identification. Briefly, the cellMCD procedure computes this likelihood criterion with a set of selected entries flagged as unusual values. The cellMCD procedure then finds the set of flagged entries that optimizes this criterion to obtain robust estimates of the center and covariance of the distribution. In our context, for each input entry $z_{gj}$, cellMCD computes a standardized residual $r_{gj}^{*}$ as a measure of the deviation of $z_{gj}$ from its expected value given the other entries on row g and the overall distribution of z-statistics. Entries with $\left|z_{gj}\right|$ exceeding a threshold (default = 2.57 = 99th percentile of a $\chi_{1}^{2}$) are flagged as outliers. The procedure is implemented in the cellWise (v. 2.5.4) R (v. 4.5.1) package on CRAN and described in detail by Raymaekers and Rousseeuw [8].

Applying the cellMCD procedure to Z yields results that prioritize specific statistical results for further study. The entries of Z that are flagged as outliers are specific statistical results that stand out from the bulk of Z entries that presumably represent the distribution of statistics for true null hypotheses. These results are fundamentally different from the results of SSz and Fisher’s method. Fisher’s method and SSz prioritize rows of Z (individual genes) for further study, while cellMCD flags individual entries of Z (associations of specific genes with specific phenotypes) for further study. In this way, cellMCD more directly addresses the objective of prioritizing specific results for further study than do Fisher’s method and SSz.

The cellMCD procedure also provides results that are useful for the objective of prioritizing specific genes (rows of Z) for further study. In particular, cellMCD computes robust estimates of center and covariance. We compute the Mahalanobis distance of each row relative to these robust estimates of center and covariance. For each gene g=1,…,m, let $h_{g}$ represent this Mahalanobis distance. Genes with the greatest Mahalanobis distances can then be prioritized for further study. We may also report each distance $h_{g}$ as a standardized criterion $h_{g}^{*}=\sqrt{h_{g}/d}$ and compute a *p*-value by comparing $h_{g}$ to a central chi-square distribution with d degrees of freedom.

## 4. A Visual Comparison of the Methods’ Significance Regions

**Figure 1:**
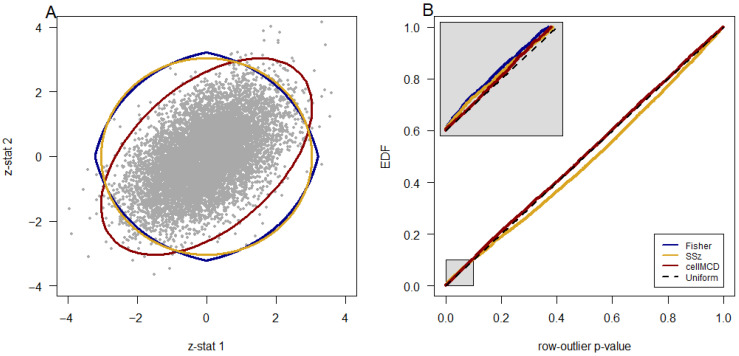
cellMCD boundaries account for correlation and scale of bivariate normal data. Panel (**A**) shows a scatterplot of 10,000 simulated points from a bivariate normal distribution with mean (0, 0), standard deviation of 1 for each variable, and correlation of 0.5 between the two variables. The estimated 99% inclusion boundaries by SSz, Fisher’s method, and cellMCD are shown in yellow, blue, and red, respectively. Panel (**B**) shows a uniform quantile–quantile plot of the row-outlier detection values of each method, with a zoom-in panel showing the region with *p* < 0.10 in greater detail.

Figure 1 illustrates these methods on a simulated bivariate normal data set without any outliers. Figure 1A shows a scatterplot of the data with the estimated 99% inclusion boundaries of each method that are intended to capture 99% of the data. The SSz method has a perfectly circular boundary, and Fisher’s method has a pointed circular boundary. These two methods do not adjust for the correlation or scale of the data. The cellMCD method draws an elliptical boundary that accounts for the correlation and scale of the data. Each method computes a row-outlier *p*-value for each point in the scatter plot. Figure 1B shows a uniform quantile–quantile plot of the *p*-values of each method. The uniform quantile–quantile plot shows the empirical distribution function (EDF) of the *p*-value against the *p*-value. A good model fit is indicated by the points falling along the line y = x. Figure 1B shows that cellMCD provides a better fit to this data than do the other two methods. The lack of fit is quantitatively summarized by the root mean squared difference (RMSD) between the *p*-values and their respective EDF values. For this data set, the RMSD of Fisher’s method, SSz, and cellMCD are 0.0286, 0.0295, and 0.004, respectively. The RMSD for cellMCD is several times smaller than that of the other two methods, indicating that cellMCD better fits this data much more accurately than do the other two methods.

## 5. Simulation Studies

We simulated multivariate normal data with outliers (*n* = 1000 rows of data) using the cellWise R package simulation function generateData (Supplementary Materials). We simulated r = 1000 replications of each of 72 settings defined by unique combinations of these parameters: dimension (*d* = 5 or 10), outlier percentage (perout = 0.01 or 0.05), outlier magnitude (gamma = 0.25 or 0.50), outlier mechanisms (rows, entries, both), and correlation structure (independent, ALYZ, A09). The A09 covariance matrix $\sum_{09}\;$ has entries $\sigma_{jk}={\left(-0.9\right)}^{\left|j-k\right|}$, and the ALYZ covariance matrix is a random covariance matrix with a condition number equal to 100. Additional details are in the Supplementary Materials. We evaluated the performance of each method by ROC-type curves and summarized the results by AUC for discriminating outlier rows or rows with outlier entries from non-outlying rows. In these simulations, we compared the cellMCD Mahalanobis distance method to Fisher’s and SSz methods. Detailed results are provided in Supplementary Table S1.

**Figure 2:**
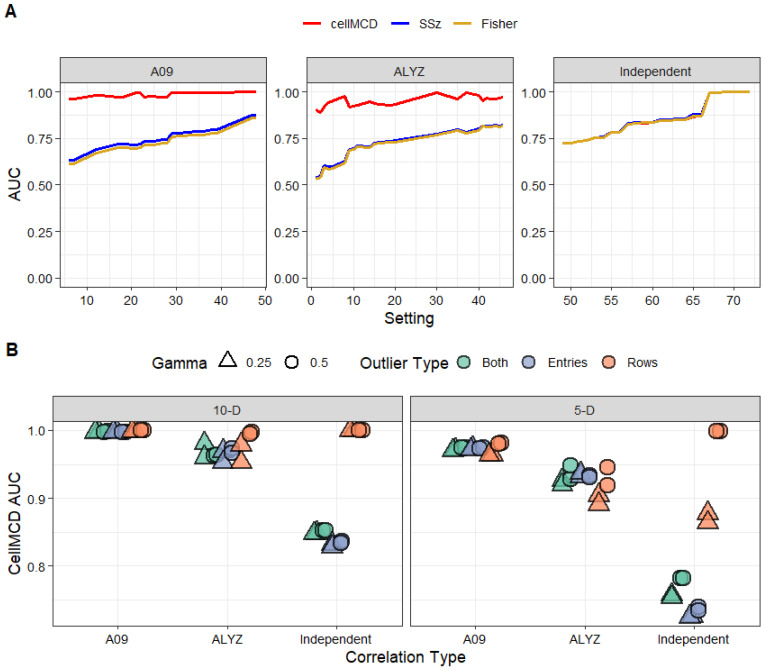
cellMCD simulation performance compared to Fisher’s method and SSz. Panel (**A**) shows a line graph of mean AUC for cellMCD (red), sum of squared Z-statistic (SSz) (blue), and Fisher’s method (gold) across settings for each correlation type (A09, ALYZ, and independent). Lines show mean AUC over *r* = 1000 replicates. Within each panel, settings are ordered by increasing standard deviation (SD) between methods to emphasize AUC agreement (low SD) or divergence (high SD). Panel (**B**) shows mean cellMCD AUC (*y*-axis) stratified by correlation type (*x*-axis) and dimension (10D, 5D). Color represents the outlier type (blue = entry outliers; orange = row outliers; green = both); shape represents outlier magnitude gamma (triangle = 0.25, circle = 0.5).

Figure 2 shows the results in a line graph. In 24 of 72 settings, the AUC of the three methods differs by less than 0.01. In the remaining 48 of 72 settings, cellMCD is clearly the best performer (Supplementary Table S1). As expected, Fisher’s and SSz’s performance almost perfectly matched in all settings (Figure 1A and Figure S1). In settings with independent association statistics for which Fisher’s method and SSz are known to be excellent, the performance of cellMCD is similar (Figure 2A). In settings with correlation, the performance of cellMCD is much better than that of Fisher’s and SSz methods (Figure 2B). All three methods were excellent at classifying rows with outlier entries under independence and performed worse at classifying data sets with both outlier rows and outlier entries (Figure 2B and Figure S1). In settings with correlation (ALYZ, A09), cellMCD achieved consistently higher AUC values than those of Fisher’s and SSz methods, particularly in scenarios with outlier rows or data sets with both outlier rows and outlier entries (Figure 2). cellMCD performance varied primarily with the number of dimensions and did not change due to the percentage or magnitude of outlier contamination (Figure 2B). Overall, patterns in AUC favor the cellMCD method, showing a higher mean AUC for cellMCD and comparable performance to classical methods for the independent settings (Figure 2).

## 6. A Pediatric Leukemia Cell Line CRISPR Drug Screen

CRISPR drug screening was performed as previously described [3,13]. We analyzed data from pooled CRISPR–Cas9 knockout screens conducted in ML-2, an AML human cell line, using a custom library targeting 2442 genes. We used the single-guide RNA (sgRNA) sequences from the Brunello library and implemented CRISPR through the all-in-one LentiCRISPRv2 vector system containing both Cas9 nuclease and sgRNAs. The library targeted each gene with four sgRNAs and included 100 non-targeting controls.

ML-2 cells were transduced with the library at a low multiplicity of infection (MOI = 0.3–0.4) to minimize the likelihood of multiple sgRNAs per cell. After puromycin selection, transduced cells were expanded and then split into control (DMSO) and drug-treated groups of cytarabine (AraC), daunorubicin (Dauno), and etoposide (Etop), with drug concentrations chosen to achieve ~30% growth inhibition (IC_30_) over seven cell doublings, ensuring sufficient genomic coverage for sequencing. Screens were performed in triplicate. Samples were collected post-puromycin selection (baseline) immediately and after seven doublings for each drug and control (final-day), followed by genomic DNA extraction, library amplification, and next-generation sequencing (Illumina NovaSeq, 100 bp single-end, Illumina, San Diego, CA, USA).

Processed reads were analyzed using the MAGeCK Robust Rank Aggregation (RRA) pipeline (v0.5.9.4) to align sgRNAs, quantify read counts, normalize guide counts, and identify gene-level depletion or enrichment under each treatment condition in final-day samples. MAGeCK-RRA [14,15] was used to aggregate abundance per gene (across guides and replicates per treatment) and to compare gene abundance between the last day for each drug vs. the last day for the control [14] (log fold-change [LFC]). Here, a positive (LFC > 0) or negative (LFC < 0) change in gene abundance was associated with a sensitivity or resistance phenotype, respectively. For each drug (column) d, we computed per-drug z-scores for genes by the usual approach of subtracting the column mean and dividing by the column standard deviation. We then applied the three procedures to the Z-matrix (Supplementary Table S2) as described above and shown in Figure S2.

**Figure 3:**
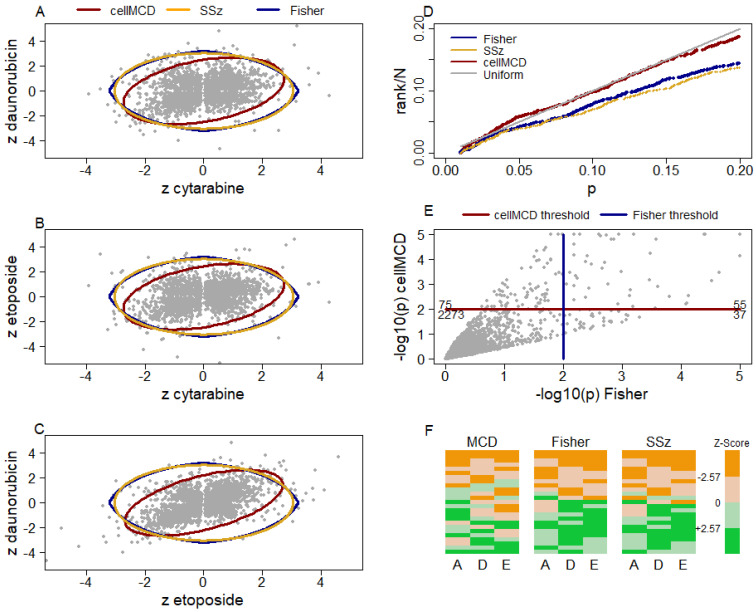
cellMCD provides more reliable outlier detection in AML CRISPR drug screen analysis. Scatterplots of z-statistics for all genes for (**A**) daunorubicin and cytarabine, (**B**) etoposide and cytarabine, and (**C**) daunorubicin and etoposide. In each scatterplot, a point represents a z-statistic for one gene’s association with the sensitivity or resistance to the two drugs named on the axis labels. The red, yellow, and blue lines represent the 99% inclusion boundaries of cellMCD, SSz, and Fisher’s method, respectively. Panel (**D**) shows a quantile–quantile plot for the non-significant row-outlier detection *p*-values (*p* > 0.01) of cellMCD (red), Fisher’s method (blue), and SSz (yellow), respectively. The gray line represents the expected quantiles of the uniform (0.01, 1) distribution. Panel (**E**) shows a scatter plot of the $-log_{10}\left(p\right)$ values of cellMCD versus those of Fisher’s method for row-outlier detection. Results with $-log_{10}\left(p\right)>5$ are shown as 5 in this figure. The horizontal red line at y = 2 corresponds to p = 0.01 by cellMCD, and the vertical blue line at x = 2 corresponds to *p* < 0.01 by Fisher’s method. Points above the red line are significant at *p* < 0.01 by cellMCD, and points to the right of the blue line are significant by Fisher’s methods. The numbers at the left and right edges of the plot provide the number of genes falling in the corresponding quadrant of the plot. Panel (**F**) shows heatmaps of the z-statistics of the 25 most significant genes by each row-outlier detection method. Rows represent genes. Columns are labeled as A for cytarabine (also known as araC), D for daunorubicin, and E for etoposide. The color scale shows z-statistic values by their sign (orange for positive and green for negative) and magnitude (less intensity for $\left|z_{gj}\right|<2.57$ and greater intensity for $\left|z_{gj}\right|>2.57$).

Figure 3 shows scatterplots of all genes’ z-statistics for daunorubicin and cytarabine (Figure 3A), etoposide and cytarabine (Figure 3B), and daunorubicin and etoposide (Figure 3C). As in the scatterplot of Figure 1A, the elliptical boundary of cellMCD better approximates the distribution of the bulk of the data in these three scatterplots than do the perfectly circular SSz boundary and pointed circular Fisher’s method boundary. A quantile–quantile plot of the three methods’ row-outlier detection *p*-values (limited to those > 0.01 to include primarily non-outlier genes) against the uniform distribution shows that cellMCD better fits the bulk of the data (and thus better identifies outliers) than do the other two methods (Figure 3D). This result and the superior performance of cellMCD in simulations with correlated data sets strongly suggest that the cellMCD results are more reliable than those of the other two methods, although definitive statements regarding the veracity of any particular result cannot be made with this real-world data. At the *p* = 0.01 level, cellMC, Fisher’s method, and SSz detected 130, 92, and 87 genes as row outliers, respectively. This is also consistent with cellMCD having better AUC than Fisher’s method and SSZ in simulations with correlated z-statistics. There were 55 genes significant by both cellMCD and Fisher’s method, 75 genes significant by cellMCD but not Fisher’s method, and 37 genes significant by Fisher’s method but not cellMCD (Figure 3E). Similarly, there were 54, 76, and 33 significant by both SSz and cellMCD, significant by cellMCD but not SSz, and significant by SSz but not cellMCD, respectively. Heatmaps of the input z-statistics for the top 25 genes by each method show that Fisher’s method and SSz find fewer genes with variation in the signs of the z-statistics across drugs than does cellMCD (Figure 3F).

The cellMCD entry-outlier detection procedure flagged individual entries as potentially interesting that were not readily apparent from the input z-statistics. cellMCD flagged 261 entries as outliers with standardized residual $\left|r_{gj}\right|>2.57$ and 162 of those entries had input z-statistic $\left|z_{gj}\right|<2.57$. Several of these unique discoveries are supported by published literature. Examples include BCL2 for cytarabine and CBFB for etoposide. BCL2 has long been associated with resistance to the standard cytarabine-based chemotherapy [16] and is the target of venetoclax, which is being investigated in multiple AML clinical trials (https://clinicaltrials.gov/search?cond=AML&intr=venetoclax (accessed on 29 September 2025)). Etoposide induces DNA breakage at the CBFB locus in hematopoietic cells [17]; CBFB is commonly mutated or fused with MYH11 in pediatric AMLs [18]. Our analysis identified other biologically relevant genes, but they were not previously known to contribute to drug resistance in AML. One example is RUNX2, which was associated with AraC and Dauno resistance in our study. RUNX1 forms a complex with CBFB, is consistently upregulated in AML [19], and has been shown to cooperate with the CBFB–MYH11 fusion protein to promote leukemogenesis in mice [20]. Our analysis found NUP98 and IDH2 to be associated with AraC resistance. NUP98 rearrangements make up a well-known subtype of AML [21]. IDH2 mutations are known to be associated with epigenetic reprogramming in AML and are frequent in adult AML [22]. WT1 is another recurrently mutated or overexpressed gene associated with poor prognosis [23] and associated with daunorubicin resistance. PIK3CG encodes the enzyme PI3Kγ, which functions as a critical enzyme for maintaining leukemia stem cell self-renewal and thus promotes AML progression [24]. and associated with etoposide sensitivity in this analysis. Finally, ERCC4/FANCQ and TOP1 were associated with etoposide resistance in our analysis. Mutations in FANCQ are associated with Fanconi anemia (FA) development [25]. FA has a high cumulative risk of developing AML [26]. While the role of TOP1 in AML is less well known, it has functional similarity to TOP2A (the target of etoposide), which suggests that TOP1 may contribute to etoposide drug response [27].

## 7. Discussion

The statistical analyses of modern multi-dimensional data sets produce association testing results that are massive and complex. It is challenging to process such results to obtain prioritized lists of genes or gene–phenotype associations to guide follow-up research. Here, we adapt Fisher’s method, the SSz meta-analysis methods, and the cellMCD multivariate outlier detection method for the problem of identifying rows of a large matrix of z-statistics that may indicate a biologically interesting (non-null) result. We use each method to compute row-outlier detection *p*-values and prioritize those rows (genes) with the smallest row-outlier detection *p*-values. We use pairwise scatterplots and uniform quantile–quantile plots of the row-outlier detection *p*-values to visually evaluate the goodness of fit of the significance boundaries of these methods to the distribution of the data.

We evaluated these approaches in simulation and in the analysis of a CRISPR drug screening study of an acute myeloid leukemia cell line across 3 drug treatment conditions. In our simulation studies, we used an area under the curve metric to quantify the performance of the methods to prioritize true row outliers from non-outliers. The three methods had practically identical performance in terms of AUC in 24 simulation settings with uncorrelated columns. In 48 settings with correlation among the columns of the z-statistics, cellMCD greatly outperformed the other two methods. Additionally, in the analysis of the CRISPR drug set, cellMCD fit the data much better than the other two methods. These results suggest that cellMCD may be a useful and reliable tool for interrogation of large matrices of z-statistics associating genes with multiple phenotypes.

Our results show that while Fisher’s and SSz may be sufficient methods to summarize association statistics across multiple phenotypes when individual tests are independent (i.e., when testing on different sets of genes), these assumptions are often violated in omic data where phenotypes and experimental contexts are often correlated. cellMCD models the full covariance structure of the z-statistic matrix, allowing it to account for correlation directly and distinguishing correlation-driven signals from outlying ones. Conceptually, whereas Fisher’s and SSz may ignore more modest, nuanced multivariate outliers, cellMCD can distinguish and accommodate multi-dimensional association structures to find both modest and strong multivariate outliers. In addition, cellMCD is especially advantageous in providing both gene-level (row) and gene-by-phenotype-level (entry) results, which are more interpretable for consensus ranking and localized outlier detection.

We found that cell MCD is a useful method to use CRISPR data to identify genes involved in sensitivity and resistance to drugs used for the treatment of AML. Several other methods have been used to evaluate other forms of omic data for evidence of gene involvement in drug resistance, drug sensitivity, and treatment outcomes in AML [28,29,30,31]. However, these methods were not designed for the evaluation of CRISPR data.

Future research should evaluate the performance of cellMCD [8] and the recently proposed cellMCD+ [9] on z-statistic matrices with many more columns to determine their reliability in more complex settings. Example applications with many more columns are already common in practice. CRISPR drug screens may evaluate multiple drugs or drug combinations in multiple cell lines. For example, a study evaluating 13 drugs in 9 cell lines would produce a matrix of z-statistics with 117 columns. A prognostic association screening study that evaluates the association of genomics, epigenomics, transcriptomics, and proteomics (four forms of omics) with three endpoints in each of three different clinical trials (nine outcome variables) would produce a matrix with 36 columns. An analysis seeking to integrate data from these two example studies would involve analyzing a matrix with 153 columns. We are optimistic that cellMCD will scale up nicely to these problems and be an effective tool for exploring and better understanding such data.

## Data Availability

The matrix of z-statistics are provided as supplementary materials file with this manuscript. The primary CRISPR profiles are not publicly available due to ongoing analyses and project restrictions. These discussed methods are available in the multi-omic multi-association z-statistics (momaz) package at https://github.com/doraaobodo/momaz accessed on 29 September 2025.

## Supplementary Materials

The following supporting information can be downloaded at https://www.mdpi.com/article/10.3390/genes17010049/s1, Figure S1: Fisher’s method and SSz simulation performance across settings; Figure S2: Schematic of CRISPR screening data preprocessing; Table S1: Results of simulation studies; Table S2: Results of ADE CRISPR drug screen analysis using celMCD, Fisher’s method, and SSz.
