# Supplementary material for: cellMCD Effectively Discovers Drug Resistance and Sensitivity Genes for Acute Myeloid Leukemia
Source: Genes (Basel). 2026 Jan 1;17(1):49. doi: 10.3390/genes17010049 (PMC12841039; doi:10.3390/genes17010049)
Supplement: Supplementary file 1 [file genes-17-00049-s001.zip › Supplementary_Materials_2025_09_30_revised.pdf]

Simulation Study Methods

We generated multivariate normal data using the cellwise R package function generateData(). We generated data for 1000 rows (genes) with mu = 0, and covariance structure Σ determined by generateCorMat(). We applied the meta-analysis methods for 1000 replicates per setting and aggregated results for comparison. For each setting, we specified:

- Dimension (d): number of columns in matrix
- Contamination rate (perout): fraction of rows or fraction of entries to contaminate.
- Outlier magnitude (gamma): distance of outliers from the robust matrix center.
- Outlier type: we chose to use 3 of the 4 available types defined as:
    - rows: The algorithm replaces a fraction of rows by point contamination in the direction of the last eigenvector of Σ. This mimics row-level anomalies aligned with a weakest-variance direction, a classical stress test for robust scatter estimators.
    - entries: first selects contaminated entries at random; within each affected row, it replaces those entries by a multiple of the smallest eigenvector of Σ restricted to those columns. This yields row-local, coherent deviations—a harder entry-wise scenario.
    - both: combines row-wise and entry-wise contamination, using half of perout for rows and the remaining half for entries.
- Sigma: We generated covariance structures based on correlation types:
    - Independent: $\Sigma = I_d$. This favors methods whose null assumptions rely on independence.
    - ALYZ: a random correlation matrix as in [1], with a prescribed condition number: $CN = \kappa(\Sigma) = \lambda_{max}/\lambda_{min}$; default 100). Larger CN produces stronger cross-column correlation. We fixed Σ once per setting so all replicates share the same correlation structure.
    - A09: a covariance structure defined by $\rho_{jk} = (-0.9)^{|k-j|}$, where $k, j \in \{1, \ldots, d\}$ index matrix column. This covariance structure produces strong alternating correlations that stress methods assuming independence.

For each replicate, we computed one per-row score from each method. We then ranked rows by each score to evaluate performance against the ground-truth label l_g where $l_g = 1$ for outliers and $l_g=0$ for non-outliers. Within each replicate and method, we ranked rows by decreasing score. Let $1\{\cdot\}$ be the indicator function that equals 1 if the enclosed statement is true and 0 if the enclosed statement is false. Let m_0 be the number of genes that are not outliers and let m_1 be the number genes that are outliers. Let $m = m_0 + m_1$. Then, let $(g)=1,\ldots,m$ index the ordered $p_{(1)} <= p_{(2)} <= \ldots <= p_{(m)}$. For each $k=1,\ldots,m$, let $x_k = \sum_{k=1}^{m} 1(l_g = 0)$ and let $y_k = \sum_{k=1}^{m} 1(l_g = 1)$ be the cumulative number of genes that are not outliers and the cumulative number genes that are outliers as indexed by the ordered p-values up through k.

We then compute the area under the curve (AUC) by the trapezoidal rule:

$$\text{AUC} \approx \frac{1}{m} \sum_{k=1}^{m-1} (x_{k+1} - x_k) \frac{y_{k+1} + y_k}{2}.$$

To summarize across replicates, we record AUC per replicate and report the mean AUC (±SD) for each setting; and (ii) linearly interpolate each ROC onto a common grid $u \in [0,1]$ (step 10^{-4}) and average the resulting curves row-wise to display mean AUC.

St. Jude - Confidential

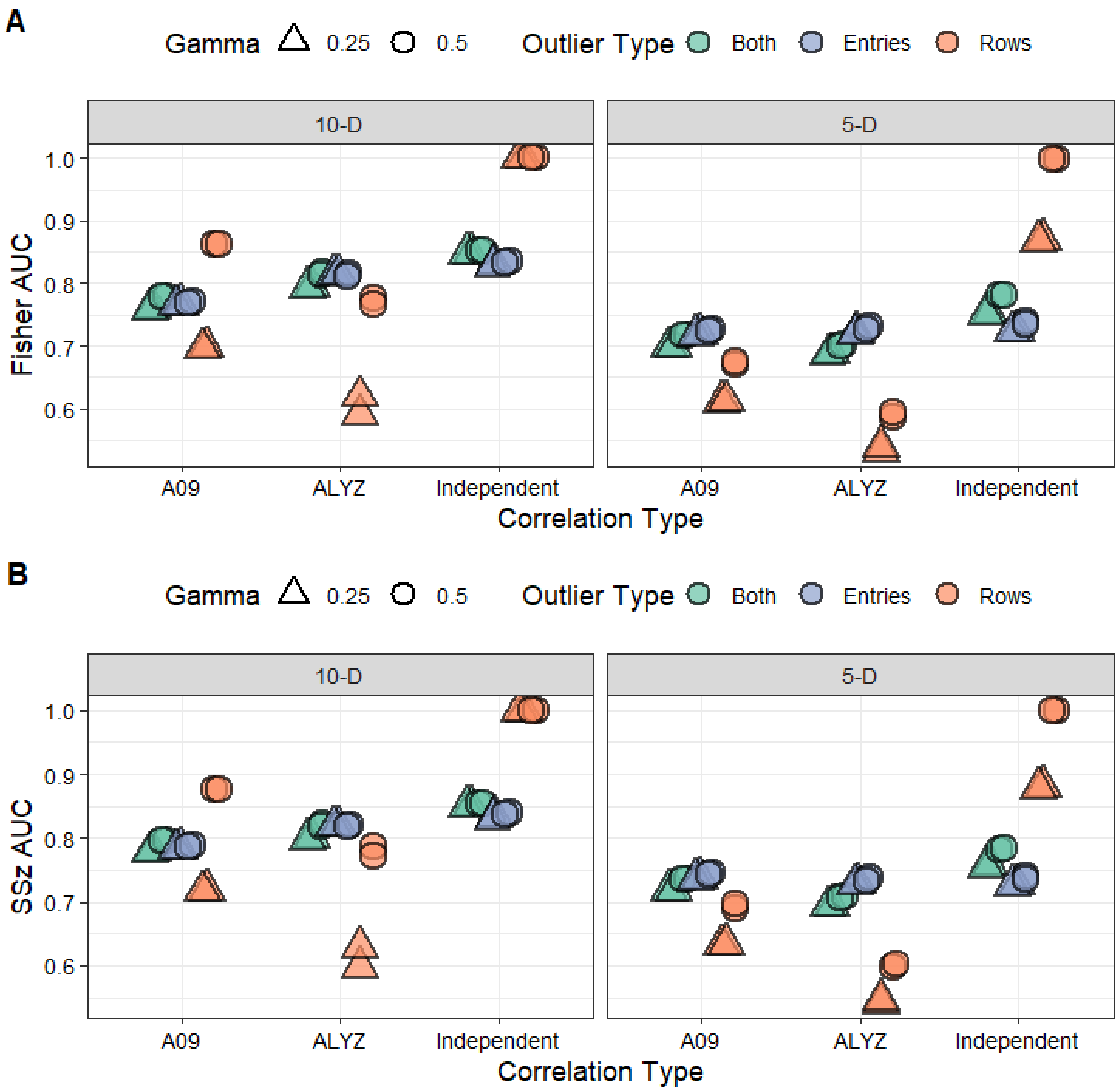

Figure S1: Fisher and SSz simulation performance across settings. Mean cellMCD AUC (y-axis) stratified by correlation type (x-axis) and dimension (panels: 10D, 5D). Color represents outlier mechanism (green = both; orange = rows; blue = entries); shape represents outlier magnitude (gamma: triangle = 0.25, circle = 0.5).

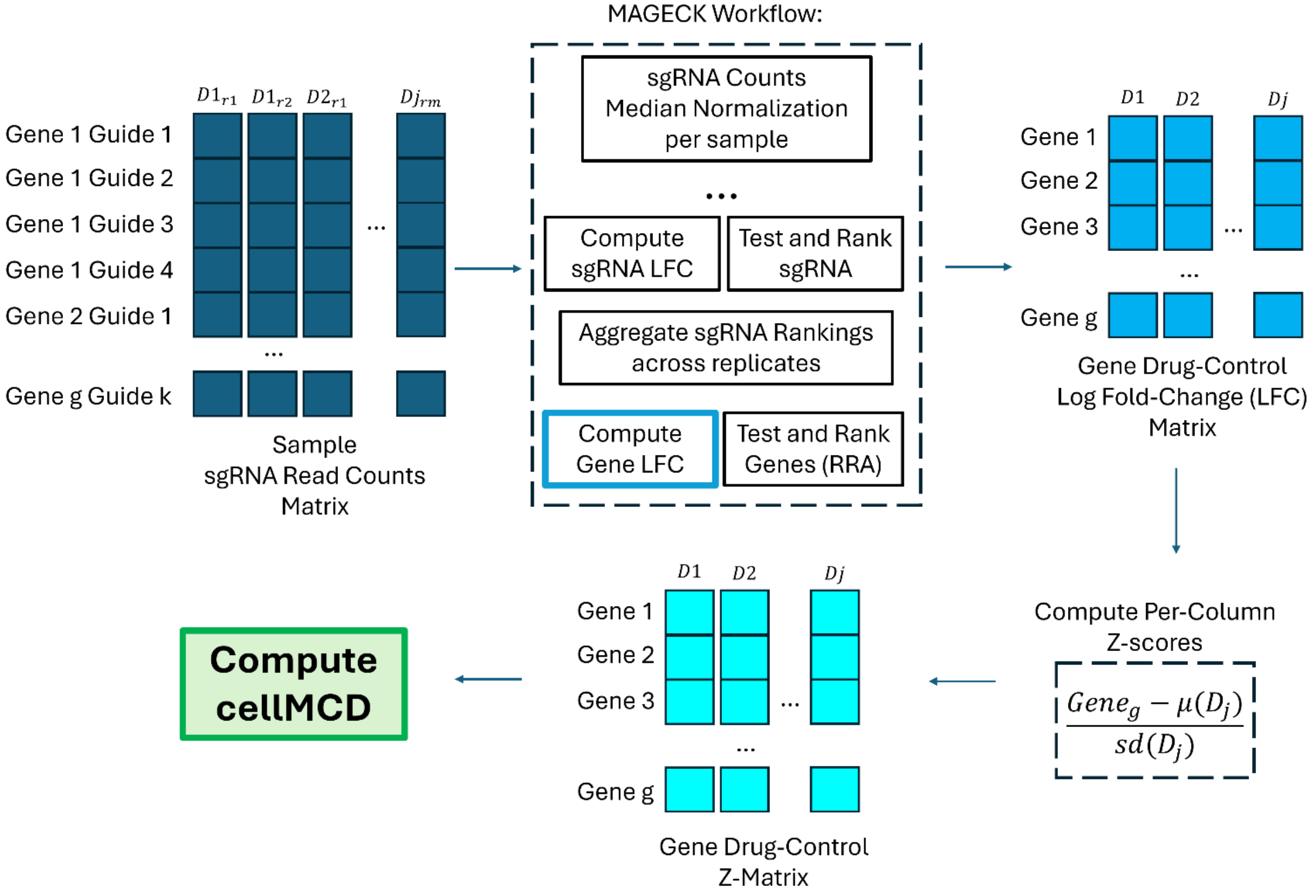

Figure S2: Schematic of CRISPR screening data preprocessing.

References:

1. Raymaekers, J.; Rousseeuw, P.J. The Cellwise Minimum Covariance Determinant Estimator. *J. Am. Stat. Assoc.* **2024**, *119*, *2610-2621. h*ttps://doi.org/10.1080/01621459.2023.2267777.
